# Is there a causal relationship between HSV-1 and pemphigus vulgaris?

**DOI:** 10.1186/s40064-015-1414-8

**Published:** 2015-12-23

**Authors:** María Elisa Vega-Memíje, Francisco Javier García-Vázquez, Juan Carlos Cuevas-González, Erika Rodríguez-Lobato, Marco Antonio Aguilar-Urbano

**Affiliations:** Department of Dermatology, Dr. Manuel Gea Gonzalez General Hospital, Calzada de Tlalpan 4800, Sección XVI Delegación Tlalpan, C.P 14080 Mexico, D.F., Mexico; Molecular Pathology Laboratory, National Institute of Pediatrics, Mexico City, Mexico; Southern Associated Pathologists “Specialists in Pathology Laboratories”, Mexico City, Mexico; Pathology Laboratory, Faculty of Dentistry, Juárez University of Durango State, Durango, Dgo Mexico

**Keywords:** HSV-1, Pemphigus vulgaris, Autoimmune bullous disease

## Abstract

**Background:**

Pemphigus vulgaris is a chronic autoimmune bullous disease, the initiation of autoimmunity has been linked to viral infections. In 1974, Krain first reported the association between herpes simplex virus and pemphigus vulgaris, since then, there have been few such studies, prompting us to examine this link.

**Findings:**

We randomly selected 15 cases of PV, the diagnosis was confirmed using hematoxylin and eosin (H&E)-stained slides—2-micron sections were deparaffinized and rehydrated to be processed by immunohistochemistry, antigen retrieval was performed with 0.1 % sodium citrate, pH 6.2, endogenous peroxidase was inactivated with 0.9 % H_2_O_2_, and washes were performed with distilled water. Finally the slides were allowed to stand for 5 min in phosphate-buffered saline (PBS). The tissues were incubated for 45 min with polyclonal anti-HSV-I, (1:150, Dako Corporation, Carpinteria, CA). The MACH 1 system was applied for 15 min to visualize the reaction using 3,3′-diaminobenzidine-H_2_O_2_ (both from Biocare Medical) as substrate under a microscope. The tissues were counterstained with Lillie-Mayer’s hematoxylin (Biocare Medical). We failed to observe positivity for HSV-1 in any of the 15 PV cases that were processed.

**Conclusions:**

It is not possible to determine whether HSV-PV is a causal relationship; most studies are case reports. Thus, we propose research studies with greater methodological weight to determine the involvement of HSV in the pathogenesis of PV and demonstrate that the relationship between HSV-1 and PV is a trigger at the beginning of the disease and has an etiologic function in its pathogenesis or that it is merely a coinfection due to the immunosuppression of patients with PV.

## Findings

Pemphigus vulgaris (PV) is a chronic autoimmune bullous disease that primarily affects both genders in adults, although some reports have indicated greater frequency in women (Rodríguez et al. 2008). This bullous dermatosis is characterized by acantholysis that is caused by the loss of cohesion of keratinocytes, mediated by autoantibodies against the intercellular antigens desmogleins 1 and 3 (Sánchez-Pérez and García-Díez 2005).

The initiation of autoimmunity has been linked to viral infections, possibly through molecular mimicry, viral infections that unmask autoimmune potential, viral superantigens that are encoded by certain viruses, polyclonal activation, and diffusion of the epitope (Ruocco et al. 2014). In 1974, Krain first reported the association between herpes simplex virus (HSV) and PV (Krain 1974); since then, there have been few such studies, prompting us to examine this link.

This study was approved by the ethics committee and research, patients signed an informed consent, we randomly selected 15 cases of PV from a histopathological diagnosis center in Mexico City. The diagnosis was confirmed using hematoxylin and eosin (H&E)-stained slides—2-micron sections were deparaffinized and rehydrated to be processed by immunohistochemistry, antigen retrieval was performed with 0.1 % sodium citrate, pH 6.2, endogenous peroxidase was inactivated with 0.9 % H_2_O_2_, and washes were performed with distilled water. Finally the slides were allowed to stand for 5 min in phosphate-buffered saline (PBS).

The tissues were incubated for 45 min with polyclonal anti-HSV-I, 1:150, dilution obtained of antibody standardization, (Dako Corporation, Carpinteria, CA); the positive control were ulcer tissues that were infectted with HSV-1, and the negative control was skin without histopathological changes and viral infection. The MACH 1 (Universal Polymer Detection system) was applied for 15 min to visualize the reaction using 3, 3′-diaminobenzidine-H_2_O_2_ (both from Biocare Medical) as substrate under a microscope. The tissues were counterstained with Lillie-Mayer’s hematoxylin (Biocare Medical). We failed to observe positivity for HSV-1 in any of the 15 PV cases that were processed.

The studies that have reported a relationship between HSV and pemphigus can be classified into 3 groups: those who emphasize that viral infection is a complication of immunosuppressive therapy; proponents who claim that HSV is a trigger before the pemphigus is present, likely with an etiologic function in its pathogenesis; and studies that were examined HSV infection in PV patients but could not find any evidence (Esmaili et al. 2010).

Some groups have suggested that in patients with bullous autoimmune disorders, immunosuppressive therapy and skin and mucosal lesions facilitate viral infections (Chiu et al. 2013), whereas others have opined that in patients with recalcitrant lesions (persisting for at least 3 months), HSV must be identified (Kalra et al. 2005), in addition to injuries that inexplicably worsen, because an opportune diagnosis of viral infections is critical for avoiding fatal consequences that might arise, such as severe herpetic hepatitis and disseminated intravascular coagulation (Hocar et al. 2009).

Brandão et al. reported a case in which various samples were analyzed by PCR; the lesion on the nose was negative for several viruses, and a lesion on the upper eyelid was positive for HSV-1, resolving only after initiating treatment with acyclovir. This group referred to other studies that support the relationship between pemphigus and HSV (Brandão et al. 2011).

In 2013, Oliveira et al. studied 23 patients with PV [15 women (65 %) and 8 men (35 %)], from whom 105 samples were collected. HSV was detected by PCR in 36 samples from 17 patients, and 6 patients were negative for the virus; the authors identified HSV infections in PV lesions that were recurrent or persistent (Oliveira-Batista et al. 2013).

Esmaili et al. examined 38 patients with pemphigus vulgaris (19 men and 19 women), 12 of whom had a history of cold sores (31.57 %). By PCR, 32 skin tissues and 5 peripheral blood samples were negative for HSV (Esmaili et al. 2010). Our results are similar, failing to not any positive cases in 15 tissues with PV by immunohistochemistry.

Based on the existing literature, it is not possible to determine whether HSV-PV is a causal relationship, regardless of the technique of identification used (immunohistochemistry or PCR); most studies are case reports. Thus, we propose research studies with greater methodological weight (cohort studies or clinical trials on age, sex, immunocompromised and other clinical variables are included) in this way we be able to determine the involvement of HSV in the pathogenesis of PV and demonstrate that the relationship between HSV-1 and PV is a trigger at the beginning of the disease and has an etiologic function in its pathogenesis or that it is merely a coinfection due to the immunosuppression of patients with PV.

## Abbreviations

PV: Pemphigus vulgaris
HSV: herpes simplex virus
H&E: hematoxylin and eosin
PBS: phosphate-buffered saline
